# Trends and Disparities in Gout-Related Mortality in the United States, 1999-2024: A Multiple-Cause-of-Death and Joinpoint Regression Analysis

**DOI:** 10.7759/cureus.112594

**Published:** 2026-07-13

**Authors:** Muhammad Uzair, Hamza B Amir, Zenab M Khan, Haashir A Siddiqi, Astad Y Sidhwa

**Affiliations:** 1 Internal Medicine, Independent Researcher, Karachi, PAK

**Keywords:** age-adjusted mortality rate, cdc wonder, covid-19 pandemic, gout, gout-related mortality, health disparities, joinpoint regression, multiple cause of death

## Abstract

Introduction

Gout is the most common inflammatory arthritis and clusters with cardiometabolic and renal disease, yet population-level trends in gout-related mortality spanning the pre-pandemic and COVID-19 periods have not been characterized. We described United States gout-related mortality from 1999 to 2024, modeled its temporal trends overall and across demographic and regional strata, and profiled co-occurring causes of death.

Methods

In a retrospective serial cross-sectional analysis of the Centers for Disease Control and Prevention (CDC) Wide-ranging Online Data for Epidemiologic Research (WONDER) Multiple Cause of Death database, we identified deaths with any mention of gout (International Classification of Diseases, Tenth Revision (ICD-10) M10.0-M10.4, M10.9) in the underlying or contributing cause-of-death fields for 1999-2024, combining the 1999-2020 bridged-race and 2021-2024 single-race files. Age-adjusted mortality rates (AAMRs), standardized to the 2000 US standard population, were analyzed with the National Cancer Institute Joinpoint Regression Program (version 6.0.1, National Cancer Institute, Bethesda, Maryland) to estimate the annual percent change (APC) per segment and the average annual percent change (AAPC) across 1999-2024. Eight prespecified co-occurring conditions were described.

Results

A total of 55,721 gout-related deaths were identified. Decedents were predominantly male (67.0%), non-Hispanic White (74.3%), and aged 75 years or older (68.1%). The overall AAMR was 0.57 per 100,000 in 1999 and 0.63 in 2024; the full-period AAPC was not significant (+0.30%; 95% confidence interval (CI), -0.46 to +1.07). The trend comprised four phases: a rise to 2011 (APC +1.03%), a decline to 2018 (APC -2.82%), a sharp rise to 2021 (APC +10.48%) concentrated in the pandemic years, and a decline thereafter (APC -4.76%). The pandemic-era rise was statistically significant for male, non-Hispanic Black, Hispanic, and Southern decedents but not for other strata; over the full period, only non-Hispanic Asian or Pacific Islander decedents (AAPC -2.72%) and the Midwest (AAPC -0.98%) showed a significant net change, both declines. Crude mortality rose steeply with age and increased from metropolitan to rural counties. Hypertension was the most frequent co-occurring condition (61.6%), followed by ischemic heart disease (36.2%), diabetes mellitus (28.8%), heart failure (26.4%), and renal failure (19.1%).

Conclusions

US gout-related mortality was stable on average between 1999 and 2024 but moved through four distinct phases, accelerated around the COVID-19 pandemic, and varied by sex, race and Hispanic origin, region, and urbanization. The trajectory tracks the cardiometabolic-renal multimorbidity that accompanies gout: these deaths are predominantly co-listed with hypertensive, cardiovascular, metabolic, and renal disease rather than attributed to gout itself. Reducing this mortality will depend less on new therapy than on delivering established gout and comorbidity care to the older, multimorbid, and underserved populations in whom these deaths are concentrated.

## Introduction

Gout is the most common form of inflammatory arthritis. It develops when sustained hyperuricemia drives the deposition of monosodium urate crystals in and around joints, and although it presents clinically as intermittent flares, it is a chronic disease [1]. In the United States, an estimated 9.2 million adults (3.9%) were living with gout in 2015-2016, including 5.2% of men and 2.7% of women, a prevalence that had held roughly steady across the preceding decade [2]. The global burden has continued to climb: an estimated 55.8 million people worldwide had gout in 2020, age-standardized prevalence had risen by 22.5% since 1990, and the total is projected to reach 95.8 million by 2050 [3]. Gout seldom occurs in isolation. It clusters with hypertension, cardiovascular disease, chronic kidney disease, and metabolic disorders [1,2], a pattern that has long raised the question of whether the disease shortens survival. Prospective cohort studies have repeatedly found that it does. In the Health Professionals Follow-Up Study, men with a history of gout had higher all-cause and coronary heart disease mortality than men without gout, independent of conventional risk factors [4]. That excess has not narrowed over time. Using serial nationwide US cohorts, McCormick and colleagues compared an early cohort (1988-1994 baseline) with a contemporary one (2007-2016 baseline) and found that the premature mortality gap for patients with gout had not closed, persisting after adjustment for serum urate and atherosclerotic cardiovascular risk factors [5]. This risk extends well beyond cardiovascular causes. In a matched cohort of more than half a million patients in the US Veterans Health Administration, the most overrepresented cause-specific mortality was genitourinary, with digestive disease deaths also elevated [6]. A 2023 meta-analysis of 11 cohort studies (14.9 million participants) reported a pooled relative risk of 1.23 for all-cause mortality, 1.29 for cardiovascular mortality, 1.24 for infection-related mortality, and 1.42 for digestive disease mortality [7].

This evidence is consistent but methodologically narrow. It rests almost entirely on cohort studies that estimate patient-level hazard ratios relative to matched comparators. Such studies establish that gout carries excess risk, yet they do not describe population-level mortality rates over calendar time, identify which segments of the population bear the heaviest burden, or show how the trajectory has changed across the pre-pandemic and pandemic periods. The population-level comorbidity profile of gout-related decedents is likewise undescribed: which conditions accompany gout on the death certificate, and whether those co-occurrences have shifted, remain open questions. A coding constraint compounds the gap. Gout is seldom recorded as the underlying cause of death, with deaths attributed instead to cardiovascular, renal, or septic endpoints and gout entered as a contributing condition; an underlying-cause analysis therefore understates the burden, whereas the multiple-cause-of-death approach, which counts any mention of a condition on the certificate, is the established method for chronic diseases of this kind [8]. To our knowledge, no prior study has applied joinpoint regression to US national death certificate data to characterize population-level gout-related mortality across demographic and regional strata over a period spanning both the pre-pandemic decade and the COVID-19 era.

We addressed this gap using the Centers for Disease Control and Prevention Wide-ranging Online Data for Epidemiologic Research (CDC WONDER) Multiple Cause of Death database, characterizing US gout-related mortality from 1999 through 2024. This study had three aims. First, we profiled the distribution of gout-related deaths across age, sex, race and Hispanic origin, urbanization, and Census region. Second, we modeled temporal trends (overall and within sex, race and Hispanic origin, and Census region strata) using joinpoint regression. Third, we described eight prespecified co-occurring causes of death (hypertensive diseases, ischemic heart disease, heart failure, cerebrovascular disease, diabetes mellitus, obesity, renal failure, and sepsis) to characterize the comorbidity profile of the decedent population. The 26-year window spans the pre-pandemic decade and the COVID-19 era, and the multiple-cause design captures gout where it appears on death certificates rather than only when it is the underlying cause. The accompanying comorbidity profile provides a broader clinical context for these deaths.

## Materials and methods

Study design and reporting

We conducted a retrospective serial cross-sectional analysis of nationwide US mortality data from 1999 through 2024. Reporting follows the Strengthening the Reporting of Observational Studies in Epidemiology (STROBE) guidelines [9] and the Reporting of Studies Conducted Using Observational Routinely Collected Health Data (RECORD) extension for studies using routinely collected health data [10]. Because the analysis used de-identified, publicly available aggregate data, institutional review board approval was not required.

Data source

Mortality data were obtained from the Centers for Disease Control and Prevention's WONDER Multiple Cause of Death (MCD) database [11]. The database compiles death certificates filed in all 50 US states and the District of Columbia by the National Vital Statistics System of the National Center for Health Statistics; US territories are outside the scope of the file [11]. Two MCD vintages were queried to span the full study period: the Multiple Cause of Death 1999-2020 file (Bridged-Race categories) for years 1999-2020 [12] and the Multiple Cause of Death 2018-2024 file (Single-Race categories) for years 2021-2024 [13]. Population denominators were the bridged-race postcensal estimates for 1999-2020 [12] and the Vintage 2024 single-race postcensal estimates for 2021-2024 [13], as served by CDC WONDER on the query date of April 20, 2026. Cause of death was coded using the World Health Organization International Classification of Diseases, Tenth Revision (ICD-10) [14].

Case definition and study population

Gout-related deaths were identified as decedents with any mention of ICD-10 codes M10.0 (idiopathic gout), M10.1 (lead-induced gout), M10.2 (drug-induced gout), M10.3 (gout due to impairment of renal function), M10.4 (other secondary gout), or M10.9 (gout, unspecified) [14] in either the underlying or contributing cause-of-death fields of the death certificate. The multiple-cause approach was adopted because gout is rarely listed as the underlying cause of death; deaths are typically attributed to cardiovascular, renal, or septic endpoints, with gout coded as a contributing condition [8], and an underlying-cause-only analysis would substantially underestimate the population mortality burden. Summing across both file vintages, 44,590 decedents (80.0%) during 1999-2020 and 11,131 (20.0%) during 2021-2024 met the case definition, yielding a total of 55,721 decedents over the full 1999-2024 period.

All ages were included in the primary trend analyses. For the descriptive age stratification, no gout-related deaths were recorded below 25 years of age, and annual counts in the 25-34-year age group were fewer than 10 and were suppressed by CDC WONDER; age-specific crude rates are therefore presented from 35-44 years upward. The 148 decedents (0.3%) in the suppressed 25-34-year age group or with age not stated are retained in the overall count but not distributed among age groups, as shown in Table 1. 

**Table 1:** Characteristics of gout-related deaths (ICD-10 M10.0–M10.4, M10.9), United States, 1999–2024 (N = 55,721) ᵃ Crude rate, deaths per 100,000 population for the full 1999–2024 period; 95% CIs by normal approximation. Crude rates are not shown for residual rows. ᵇ Includes non-Hispanic American Indian or Alaska Native, more-than-one-race, and not-stated decedents; insufficient annual counts for stratified trend analysis. ᶜ Decedents aged 25–34 years (annual cell counts <10, suppressed by CDC WONDER) and decedents with age not stated, which CDC WONDER includes in overall counts but does not distribute among age groups. Each characteristic block sums to the overall total of 55,721. CI: confidence interval, ICD-10: International Classification of Diseases, Tenth Revision, CDC: Centers for Disease Control and Prevention, WONDER: Wide-ranging Online Data for Epidemiologic Research.

Characteristic	Deaths, n	%	Crude rate (95% CI)ᵃ
Overall	55,721	100.0	—
Sex			
Male	37,336	67.0	0.94 (0.93–0.95)
Female	18,385	33.0	0.45 (0.44–0.45)
Race and Hispanic origin			
Non-Hispanic White	41,389	74.3	0.80 (0.79–0.81)
Non-Hispanic Black	9,025	16.2	0.87 (0.86–0.89)
Hispanic or Latino	1,924	3.5	0.15 (0.15–0.16)
Non-Hispanic Asian or Pacific Islander	2,900	5.2	0.66 (0.64–0.68)
Other or unknownᵇ	483	0.9	—
US Census region			
Northeast	10,018	18.0	0.69 (0.68–0.71)
Midwest	13,377	24.0	0.77 (0.75–0.78)
South	19,264	34.6	0.64 (0.63–0.65)
West	13,062	23.4	0.69 (0.68–0.70)
Age group, years			
35-44	446	0.8	0.04 (0.04-0.05)
45-54	1,873	3.4	0.17 (0.16-0.18)
55-64	5,223	9.4	0.56 (0.54-0.57)
65-74	10,088	18.1	1.56 (1.53-1.59)
75-84	17,407	31.2	4.71 (4.64-4.78)
≥85	20,536	36.9	14.20 (14.01-14.40)
25-34 years or not statedᶜ	148	0.3	—
Urbanization			
Large central metro	13,083	23.5	0.60 (0.59-0.61)
Large fringe metro	11,822	21.2	0.68 (0.67-0.69)
Medium metro	12,132	21.8	0.83 (0.81-0.84)
Small metro	6,304	11.3	0.97 (0.95-0.99)
Micropolitan (nonmetro)	6,911	12.4	1.12 (1.09-1.15)
Noncore (nonmetro)	5,469	9.8	1.26 (1.22-1.29)

Stratification

Trends were analyzed overall and stratified by sex (male, female), race and Hispanic origin (non-Hispanic White, non-Hispanic Black or African American, Hispanic or Latino, non-Hispanic Asian or Pacific Islander), and US Census region (Northeast, Midwest, South, West) [15]. Three of the four race and Hispanic origin categories, non-Hispanic White, non-Hispanic Black, and Hispanic or Latino, were directly comparable between the 1999-2020 Bridged-Race [12] and 2021-2024 Single-Race [13] files. For the non-Hispanic Asian or Pacific Islander group, the bridged-race Asian or Pacific Islander category was used directly for 1999-2020. For 2021-2024, the death count combines single-race Asian and Native Hawaiian or Other Pacific Islander decedents, but the age-adjusted rates used in the trend analysis reflect single-race Asian decedents only because combined rates were not available at the time of analysis; Native Hawaiian or Other Pacific Islander decedents account for approximately 7% of the group's 2021-2024 deaths, so the most recent rates are modestly understated. This is acknowledged as a limitation. Non-Hispanic American Indian or Alaska Native, more-than-one-race, and not-stated decedents had insufficient annual death counts for stable joinpoint modeling and were not analyzed in the trend analysis but are retained in the overall count as a residual category (483 decedents; Table 1).

Rate calculation

Age-adjusted mortality rates (AAMRs) were expressed per 100,000 population and directly standardized to the 2000 US standard population [16]; AAMRs and 95% confidence intervals were as reported by CDC WONDER [11]. Crude rates for the descriptive characteristics and comorbidity analyses were computed as deaths per 100,000 population across the full 1999-2024 period, with 95% confidence intervals derived by normal approximation. CDC WONDER suppresses cells containing fewer than 10 deaths and flags rates based on fewer than 20 deaths as unreliable [11]. In the 11 stratum-specific time series used for joinpoint trend analysis and the eight comorbidity time series, every annual cell contained at least 10 deaths, all series included complete annual observations from 1999 through 2024 (n = 26 years per series), and no annual values were excluded as unreliable. For the descriptive age stratification only, suppression affected the 25-34-year age group as described above.

Joinpoint regression

Temporal trends were modeled using the National Cancer Institute Joinpoint Regression Program (version 6.0.1, National Cancer Institute, Bethesda, Maryland) [17], fitted as a log-linear model with uncorrelated errors and standard errors from CDC WONDER. The optimal number of joinpoints was selected by Monte Carlo permutation testing with 4,499 permutations per test and Bonferroni-corrected significance levels for the sequential testing procedure (overall α = 0.05) [18], with a minimum of zero and a maximum of four joinpoints, a minimum of two observations from a joinpoint to the nearest data endpoint, and a minimum of two observations between adjacent joinpoints (grid search; random seed 7160). The annual percent change (APC) was estimated for each segment between joinpoints, and the average annual percent change (AAPC) over the full 1999-2024 period was computed as a geometric weighted average of the segment APCs, with parametric 95% confidence intervals [19]. These settings follow the NCI Joinpoint program's default and recommended specifications for a series of this length: a maximum of four joinpoints is appropriate for 26 annual observations, the permutation-based selection with Bonferroni adjustment controls the overall type I error rate across the sequential tests, and the minimum-observation constraints prevent segments from being fit to too few points [17,18,19]. Eleven separate joinpoint runs were performed corresponding to the strata above. Tests were two-sided; APCs and AAPCs were considered significantly different from zero when the 95% confidence interval excluded zero.

Comorbidity analysis

Eight clinically relevant co-occurring conditions were prespecified: hypertensive diseases (I10-I15), ischemic heart disease (I20-I25), heart failure (I50), cerebrovascular disease (I60-I69), diabetes mellitus (E10-E14), obesity (E66), renal failure (N17-N19), and sepsis (A40-A41) [14]. For each condition, deaths were extracted in which the gout codes (M10.0, M10.1, M10.2, M10.3, M10.4, or M10.9) and the comorbidity code(s) appeared anywhere on the death certificate. Co-occurrence counts, the proportion of the 55,721 gout-related deaths, period crude rates per 100,000 population with 95% confidence intervals, and AAMRs with 95% confidence intervals at three reference years (1999, 2019, 2024), chosen to distinguish pre-COVID-19 from COVID-19-era trajectories, are reported. Because a single decedent may contribute to more than one comorbidity, the proportions do not sum to 100%. Consistent with convention in published CDC WONDER multiple-cause-of-death analyses, joinpoint regression was not applied to the comorbidity time series; comorbidities are presented as descriptive context for the gout-decedent population.

Software and reproducibility

CDC WONDER queries and exact parameters were documented for reproducibility. Joinpoint regression was performed in NCI Joinpoint v6.0.1 [17]; input files and session export files are available from the corresponding author on reasonable request. Trend figures were generated in Python 3.11 with Matplotlib, and tables were prepared in Microsoft Excel from the Joinpoint export files. AAMRs, APCs, and AAPCs are reported to two decimal places; crude rates to two decimal places, except the co-occurring-condition crude rates in Table 3, which are reported to three decimal places given their lower magnitude; p-values to three decimal places or as <0.001.

## Results

Characteristics of gout-related deaths

A total of 55,721 gout-related deaths (ICD-10 M10.0-M10.4, M10.9) were identified in the United States from 1999 through 2024. Decedent characteristics are summarized in Table 1. Deaths occurred predominantly in male decedents (37,336; 67.0%) compared with female decedents (18,385; 33.0%), corresponding to period crude rates of 0.94 and 0.45 per 100,000 population, respectively. By race and Hispanic origin, non-Hispanic White decedents accounted for the largest share (41,389; 74.3%), followed by non-Hispanic Black (9,025; 16.2%), non-Hispanic Asian or Pacific Islander (2,900; 5.2%), and Hispanic or Latino decedents (1,924; 3.5%); 483 decedents (0.9%) were of other or unknown race and ethnicity. Period crude rates were highest among non-Hispanic Black (0.87) and non-Hispanic White (0.80) decedents and lowest among Hispanic decedents (0.15). By Census region, the South contributed the most deaths (19,264; 34.6%) and the Northeast the fewest (10,018; 18.0%).

Gout-related deaths were concentrated at older ages, with decedents aged 85 years or older accounting for 20,536 deaths (36.9%) and those aged 75-84 years for 17,407 (31.2%). The crude mortality rate rose steeply and monotonically with age, from 0.04 per 100,000 at ages 35-44 years to 0.56 at 55-64 years, 1.56 at 65-74 years, 4.71 at 75-84 years, and 14.20 at 85 years or older. A metropolitan-nonmetropolitan gradient was also evident, with the crude rate lowest in large central metropolitan areas (0.60) and highest in noncore nonmetropolitan areas (1.26). As shown in Table 1, a residual category was retained within the race and ethnicity and age strata so that each characteristic reconciled to the total of 55,721 deaths: 483 decedents of other or unknown race and ethnicity, and 148 decedents aged 25-34 years (cells suppressed by CDC WONDER) or with age not stated.

Overall national mortality trend

**Figure 1:**
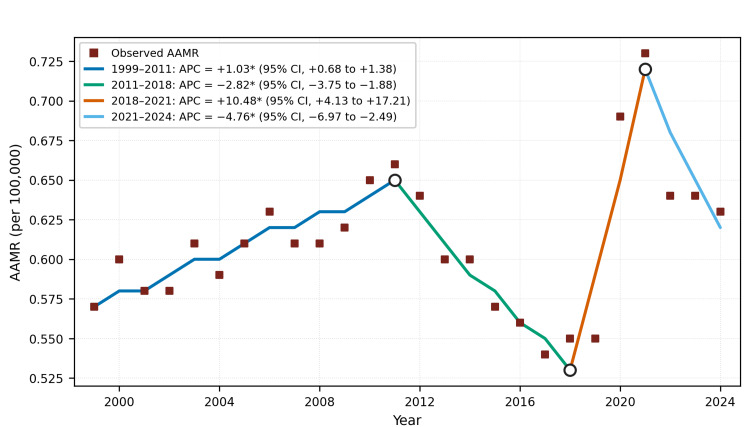
Age-adjusted mortality rates (AAMR) for gout-related deaths, United States, 1999-2024 Age-adjusted mortality rate for gout-related deaths (ICD-10 M10.0–M10.4, M10.9), United States, 1999–2024, with the fitted joinpoint model. Squares denote observed annual age-adjusted mortality rates (per 100,000, 2000 US standard population); the solid line is the fitted joinpoint trend; open circles mark the joinpoints (2011, 2018, 2021). Segment annual percent change (APC) values are shown; an asterisk denotes an APC significantly different from zero (α = 0.05).

The overall age-adjusted mortality rate (AAMR) for gout-related deaths was 0.57 per 100,000 in 1999 and 0.63 in 2024 (Figure 1). The average annual percent change (AAPC) over the full study period was +0.30% (95% CI, −0.46 to +1.07) and was not statistically significant. This flat full-period summary, however, concealed four distinct trend phases. The joinpoint model selected three joinpoints, at 2011, 2018, and 2021, defining four segments: the AAMR rose significantly from 1999 to 2011 (APC, +1.03%; 95% CI, +0.68 to +1.38), declined significantly from 2011 to 2018 (APC, −2.82%; 95% CI, −3.75 to −1.88), rose sharply from 2018 to 2021 (APC, +10.48%; 95% CI, +4.13 to +17.21), and declined significantly from 2021 to 2024 (APC, −4.76%; 95% CI, −6.97 to −2.49). All four segment APCs differed significantly from zero.

Trends by sex, race and Hispanic origin, and census region

**Figure 2:**
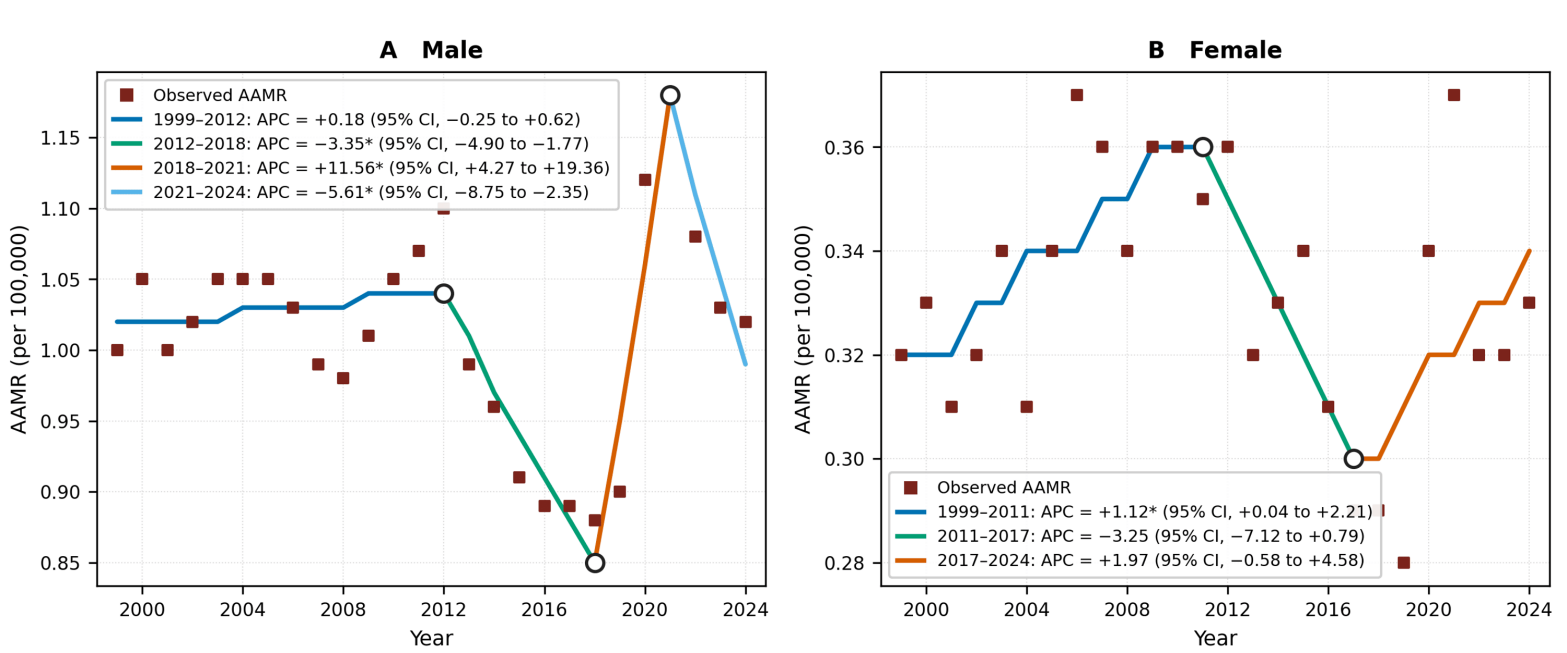
Age-adjusted mortality rates (AAMR) for gout-related deaths by sex, United States, 1999-2024 Age-adjusted gout-related mortality by sex, United States, 1999–2024, with fitted joinpoint models. (A) Male; (B) female. Squares, observed age-adjusted mortality rates; solid line, fitted trend; open circles, joinpoints. An asterisk denotes an annual percent change (APC) significantly different from zero (α = 0.05).

**Figure 3:**
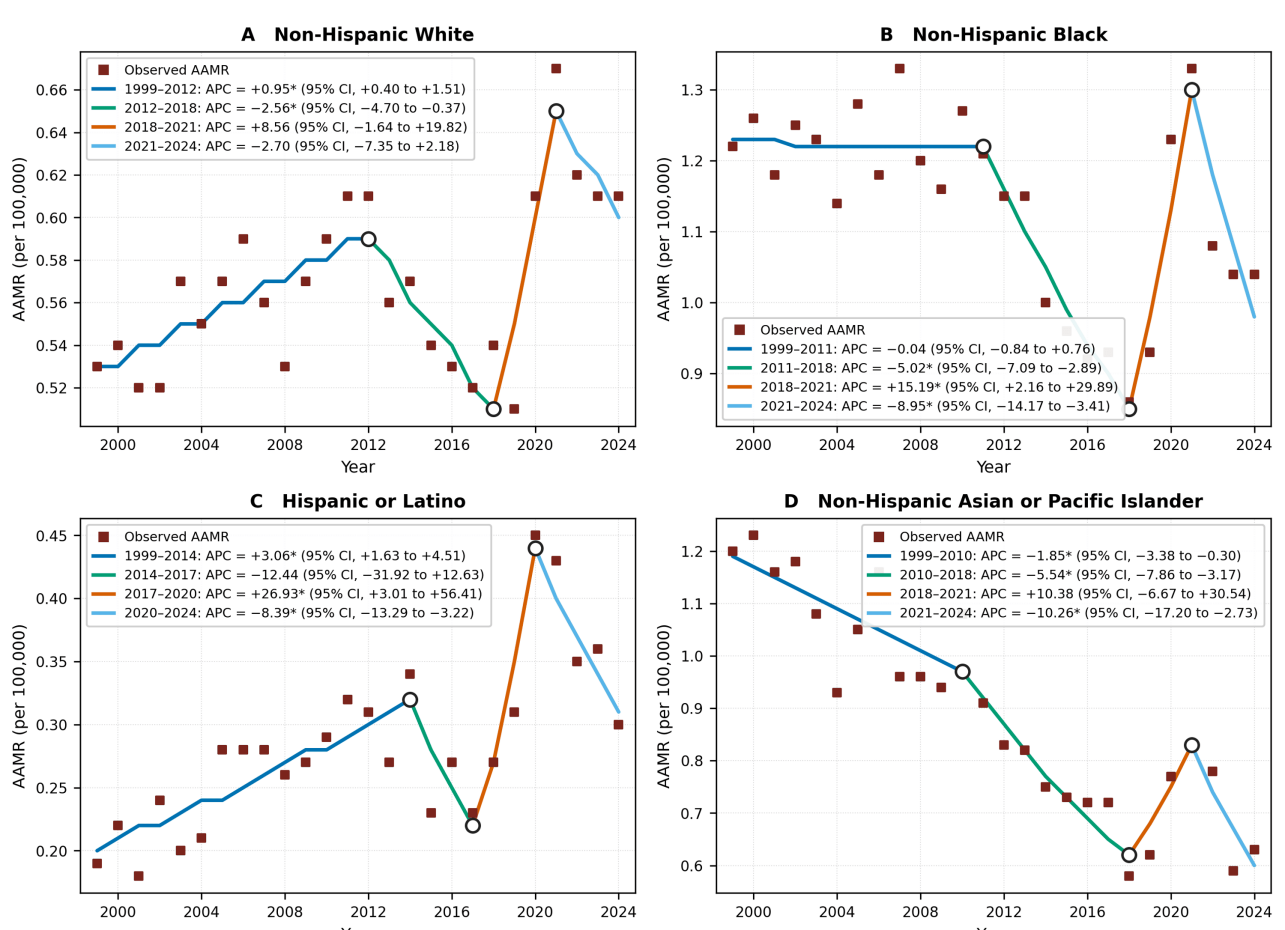
Age-adjusted mortality rates (AAMR) for gout-related deaths by race and Hispanic origin, United States, 1999-2024 Age-adjusted gout-related mortality by race and Hispanic origin, United States, 1999–2024, with fitted joinpoint models. (A) Non-Hispanic White; (B) non-Hispanic Black; (C) Hispanic or Latino; (D) non-Hispanic Asian or Pacific Islander. Squares, observed age-adjusted mortality rates; solid line, fitted trend; open circles, joinpoints. In panel D, the 2021–2024 rates reflect single-race Asian decedents only (see Methods). An asterisk denotes an APC significantly different from zero (α = 0.05).

**Figure 4:**
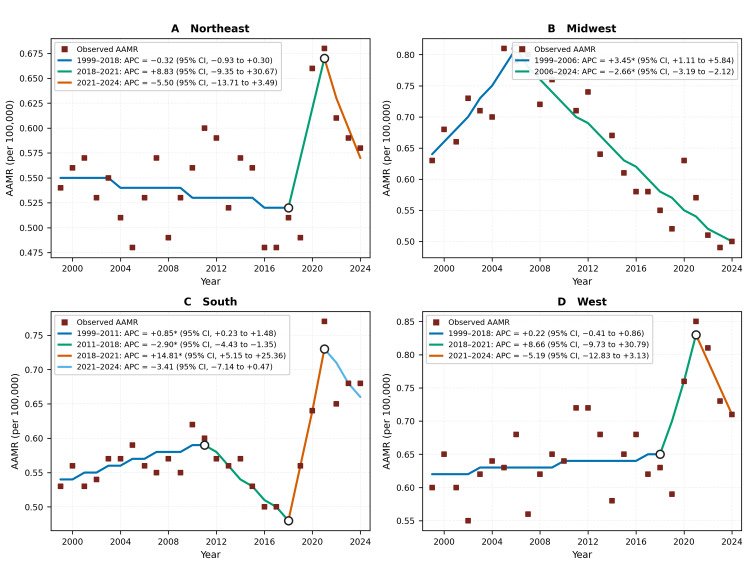
Age-adjusted mortality rates (AAMR) for gout-related deaths by US Census region, 1999-2024 Age-adjusted gout-related mortality by US Census region, 1999–2024, with fitted joinpoint models. (A) Northeast; (B) Midwest; (C) South; (D) West. Squares, observed age-adjusted mortality rates; solid line, fitted trend; open circles, joinpoints. An asterisk denotes an APC significantly different from zero (α = 0.05).

Stratified joinpoint results for all 11 strata are presented in Table 2, with fitted trends shown in Figure 2 (sex), Figure 3 (race and Hispanic origin), and Figure 4 (Census region). The four-phase trajectory identified in the overall analysis was the dominant pattern across strata, but its components reached statistical significance unevenly. The pandemic-era acceleration centered on 2018-2021 was the most variable feature: a significant rising segment was identified for the overall population, male decedents, non-Hispanic Black decedents, Hispanic decedents (2017-2020), and the South, but not for female decedents, non-Hispanic White decedents, non-Hispanic Asian or Pacific Islander decedents, the Northeast, or the West; no pandemic-era segment was selected for the Midwest. Over the full 1999-2024 period, only non-Hispanic Asian or Pacific Islander decedents (AAPC, −2.72%; 95% CI, −4.91 to −0.49) and the Midwest (AAPC, −0.98%; 95% CI, −1.68 to −0.28) had statistically significant AAPCs, both indicating a net decline (Table 2). 

**Table 2:** Joinpoint regression analysis of age-adjusted mortality rates for gout-related deaths (ICD-10 M10.0–M10.4, M10.9) in the United States, 1999–2024, by sex, race and Hispanic origin, and US Census region *APC or AAPC significantly different from zero (P < 0.05; 95% CI excludes zero). AAMR values are reported to two decimal places; APC, AAPC, and their 95% CIs to two decimal places; P-values to three decimal places. Joinpoint years are listed against the segment they begin. Joinpoint regression was performed with the NCI Joinpoint Regression Program, version 6.0.1 (log-linear model; uncorrelated errors; grid search; Monte Carlo permutation test, 4,499 permutations; parametric 95% CIs; minimum 0/maximum 4 joinpoints). AAMR, age-adjusted mortality rate (per 100,000, 2000 US standard population); APC, annual percent change; AAPC, average annual percent change; CI, confidence interval.

Stratum	AAMR 1999	AAMR 2024	Trend period	APC, %	APC 95% CI	P-value	Joinpoint year	Joinpoint 95% CI	AAPC, %	AAPC 95% CI	P-value
Overall	0.57	0.63	1999-2011	+1.03*	+0.68 to +1.38	<0.001	2011	2008-2014	+0.30	−0.46 to +1.07	0.434
			2011-2018	−2.82*	−3.75 to −1.88	<0.001	2018	2016-2019			
			2018-2021	+10.48*	+4.13 to +17.21	0.003	2021	2020-2022			
			2021-2024	−4.76*	−6.97 to −2.49	<0.001					
Sex											
Male	1.00	1.02	1999-2012	+0.18	−0.25 to +0.62	0.375	2012	2009-2015	−0.10	−1.03 to +0.83	0.833
			2012-2018	−3.35*	−4.90 to −1.77	<0.001	2018	2016-2019			
			2018-2021	+11.56*	+4.27 to +19.36	0.004	2021	2020-2022			
			2021-2024	−5.61*	−8.75 to −2.35	0.002					
Female	0.32	0.33	1999-2011	+1.12*	+0.04 to +2.21	0.043	2011	2004-2016	+0.29	−0.94 to +1.53	0.647
			2011-2017	−3.25	−7.12 to +0.79	0.107	2017	2012-2020			
			2017-2024	+1.97	−0.58 to +4.58	0.123					
Race and Hispanic origin											
Non-Hispanic White	0.53	0.61	1999-2012	+0.95*	+0.40 to +1.51	0.002	2012	2008-2016	+0.53	−0.80 to +1.88	0.438
			2012-2018	−2.56*	−4.70 to −0.37	0.025	2018	2015-2019			
			2018-2021	+8.56	−1.64 to +19.82	0.096	2021	2019-2022			
			2021-2024	−2.70	−7.35 to +2.18	0.252					
Non-Hispanic Black	1.22	1.04	1999-2011	−0.04	−0.84 to +0.76	0.914	2011	2006-2014	−0.89	−2.48 to +0.74	0.283
			2011-2018	−5.02*	−7.09 to −2.89	<0.001	2018	2016-2019			
			2018-2021	+15.19*	+2.16 to +29.89	0.024	2021	2020-2022			
			2021-2024	−8.95*	−14.17 to −3.41	0.004					
Hispanic or Latino	0.19	0.30	1999-2014	+3.06*	+1.63 to +4.51	<0.001	2014	2007-2015	+1.69	−2.08 to +5.60	0.385
			2014-2017	−12.44	−31.92 to +12.63	0.278	2017	2016-2019			
			2017-2020	+26.93*	+3.01 to +56.41	0.028	2020	2019-2022			
			2020-2024	−8.39*	−13.29 to −3.22	0.004					
Non-Hispanic Asian or Pacific Islander	1.20	0.63	1999-2010	−1.85*	−3.38 to −0.30	0.023	2010	2001-2016	−2.72*	−4.91 to −0.49	0.017
			2010-2018	−5.54*	−7.86 to −3.17	<0.001	2018	2016-2019			
			2018-2021	+10.38	−6.67 to +30.54	0.229	2021	2020-2022			
			2021-2024	−10.26*	−17.20 to −2.73	0.012					
US Census region											
Northeast	0.54	0.58	1999-2018	−0.32	−0.93 to +0.30	0.289	2018	2015-2019	+0.09	−2.21 to +2.45	0.937
			2018-2021	+8.83	−9.35 to +30.67	0.344	2021	2019-2022			
			2021-2024	−5.50	−13.71 to +3.49	0.207					
Midwest	0.63	0.50	1999-2006	+3.45*	+1.11 to +5.84	0.006	2006	2004-2009	−0.98*	−1.68 to −0.28	0.006
			2006-2024	−2.66*	−3.19 to −2.12	<0.001					
South	0.53	0.68	1999-2011	+0.85*	+0.23 to +1.48	0.011	2011	2007-2016	+0.83	−0.35 to +2.01	0.168
			2011-2018	−2.90*	−4.43 to −1.35	0.001	2018	2017-2019			
			2018-2021	+14.81*	+5.15 to +25.36	0.004	2021	2020-2022			
			2021-2024	−3.41	−7.14 to +0.47	0.080					
West	0.60	0.71	1999-2018	+0.22	−0.41 to +0.86	0.472	2018	2015-2019	+0.53	−1.78 to +2.89	0.657
			2018-2021	+8.66	−9.73 to +30.79	0.359	2021	2020-2022			
			2021-2024	−5.19	−12.83 to +3.13	0.200					

By sex (Figure 2), male gout-related mortality followed the four-phase pattern, with a non-significant change from 1999 to 2012, a significant decline from 2012 to 2018 (APC, −3.35%), a significant rise from 2018 to 2021 (APC, +11.56%), and a significant decline from 2021 to 2024 (APC, −5.61%). Female mortality followed a different two-joinpoint pattern, with joinpoints at 2011 and 2017; only the first segment was significant (1999-2011: APC, +1.12%), and neither the 2011-2017 decline nor the 2017-2024 rise reached significance. The full-period AAPC was non-significant for both sexes (male, −0.10%; female, +0.29%).

By race and Hispanic origin (Figure 3), non-Hispanic White decedents had a significant rise from 1999 to 2012 (APC, +0.95%) and a significant decline from 2012 to 2018 (APC, −2.56%); the subsequent 2018-2021 rise (APC, +8.56%) and 2021-2024 decline (APC, −2.70%) were both non-significant. Non-Hispanic Black decedents had a non-significant trend from 1999 to 2011, a significant decline from 2011 to 2018 (APC, −5.02%), a significant rise from 2018 to 2021 (APC, +15.19%, the largest 2018-2021 segment among the strata analyzed), and a significant decline from 2021 to 2024 (APC, −8.95%). Hispanic decedents had joinpoints at 2014, 2017, and 2020, with a significant rise from 1999 to 2014 (APC, +3.06%), a non-significant decline from 2014 to 2017, a significant and steep rise from 2017 to 2020 (APC, +26.93%, the largest single-segment APC observed in any stratum), and a significant decline from 2020 to 2024 (APC, −8.39%). Non-Hispanic Asian or Pacific Islander decedents declined significantly from 1999 to 2010 (APC, −1.85%) and from 2010 to 2018 (APC, −5.54%); the 2018-2021 rise was non-significant, and the 2021-2024 decline was significant (APC, −10.26%). For this group, the 2021-2024 age-adjusted rates reflect single-race Asian decedents only; Native Hawaiian or Other Pacific Islander decedents, who account for approximately 7% of the group's deaths in those years, are included in the death count but not in these rates (see Methods).

By Census region (Figure 4), the South displayed the full four-phase pattern, with a significant rise from 1999 to 2011 (APC, +0.85%), a significant decline from 2011 to 2018 (APC, −2.90%), a significant rise from 2018 to 2021 (APC, +14.81%), and a non-significant decline from 2021 to 2024 (APC, −3.41%). The Midwest had the simplest model, with a single joinpoint at 2006, comprising a significant rise from 1999 to 2006 (APC, +3.45%) followed by a significant decline from 2006 to 2024 (APC, −2.66%). The Northeast and the West each had two joinpoints, and no individual segment in either region reached statistical significance.

Co-occurring conditions

Among the 55,721 gout-related deaths, hypertension was the most frequently co-occurring condition, recorded on 34,316 death certificates (61.6%), followed by ischemic heart disease (20,185; 36.2%), diabetes mellitus (16,050; 28.8%), heart failure (14,700; 26.4%), renal failure (10,632; 19.1%), cerebrovascular disease (6,869; 12.3%), sepsis (3,699; 6.6%), and obesity (2,816; 5.1%); these counts and proportions are detailed in Table 3. Because a single death certificate may list more than one co-occurring condition, these categories were not mutually exclusive.

**Table 3:** Co-occurring causes of death listed with gout (ICD-10 M10.0–M10.4, M10.9) on US death certificates, 1999–2024 Each row reports deaths with both the gout codes (M10.0–M10.4, M10.9) and the listed condition recorded as multiple causes of death on the same certificate. Rows do not sum to the 55,721 gout-related deaths because a certificate may list more than one co-occurring condition; percentages are N / 55,721 × 100. Conditions are ordered by frequency of co-occurrence. Crude rate, deaths per 100,000 population for the full 1999–2024 period (95% CIs by normal approximation). AAMRs and 95% CIs are as reported by CDC WONDER, standardized to the 2000 US standard population. ᵃ The renal failure (N17–N19) age-adjusted mortality rate series is affected by a 2011 change in the coding of chronic renal failure, which produced an artifactual discontinuity; its temporal trend is not interpreted [20]. AAMR, age-adjusted mortality rate; CI, confidence interval; ICD-10, International Classification of Diseases, Tenth Revision.

Co-occurring condition	ICD-10 code(s)	Deaths with both, No. (%)	Crude rate per 100,000 (95% CI)	AAMR 1999 (95% CI)	AAMR 2019 (95% CI)	AAMR 2024 (95% CI)	Absolute change, 1999–2024
Hypertension	I10–I15	34,316 (61.6)	0.424 (0.420–0.429)	0.16 (0.14–0.17)	0.35 (0.33–0.37)	0.43 (0.41–0.45)	+0.27
Ischemic heart disease	I20–I25	20,185 (36.2)	0.250 (0.246–0.253)	0.26 (0.24–0.28)	0.19 (0.17–0.20)	0.17 (0.16–0.19)	−0.09
Diabetes mellitus	E10–E14	16,050 (28.8)	0.198 (0.195–0.202)	0.12 (0.10–0.13)	0.19 (0.18–0.21)	0.20 (0.19–0.22)	+0.08
Heart failure	I50	14,700 (26.4)	0.182 (0.179–0.185)	0.15 (0.14–0.17)	0.15 (0.14–0.17)	0.15 (0.13–0.16)	0.00
Renal failureᵃ	N17–N19	10,632 (19.1)	0.131 (0.129–0.134)	0.13 (0.12–0.15)	0.05 (0.04–0.06)	0.08 (0.07–0.08)	−0.05
Cerebrovascular disease	I60–I69	6,869 (12.3)	0.085 (0.083–0.087)	0.08 (0.07–0.09)	0.05 (0.05–0.06)	0.07 (0.06–0.08)	−0.01
Sepsis	A40–A41	3,699 (6.6)	0.046 (0.044–0.047)	0.02 (0.02–0.03)	0.04 (0.03–0.05)	0.05 (0.04–0.05)	+0.03
Obesity	E66	2,816 (5.1)	0.035 (0.034–0.036)	0.02 (0.01–0.03)	0.03 (0.02–0.03)	0.05 (0.04–0.06)	+0.03

**Figure 5:**
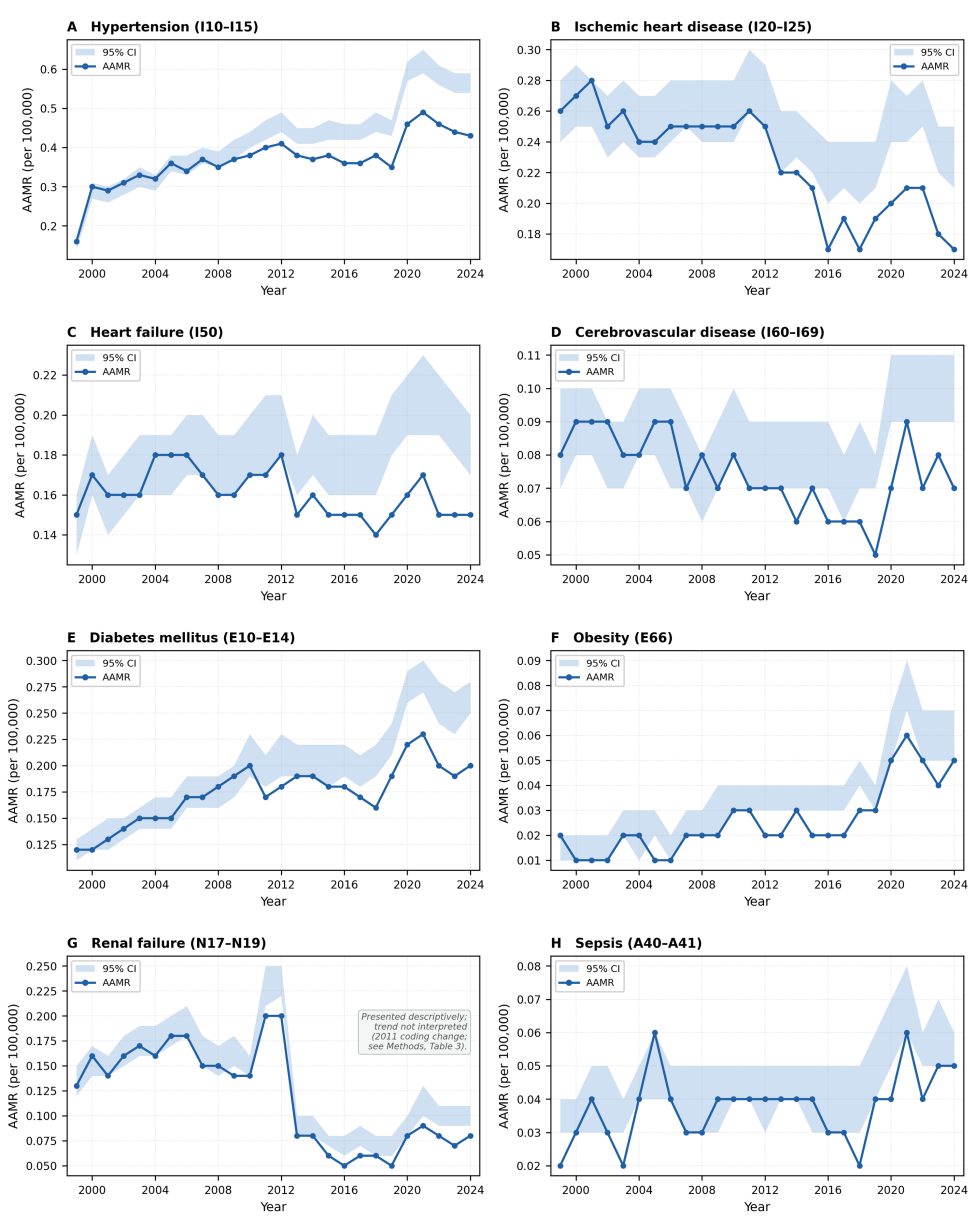
Age-adjusted mortality rates for co-occurring causes of death listed with gout (ICD-10 M10.0-M10.4, M10.9) on death certificates, United States, 1999-2024 Annual age-adjusted mortality rates for co-occurring causes of death listed with gout (ICD-10 M10.0–M10.4, M10.9) on US death certificates, 1999–2024. (A) Hypertension (I10–I15); (B) ischemic heart disease (I20–I25); (C) heart failure (I50); (D) cerebrovascular disease (I60–I69); (E) diabetes mellitus (E10–E14); (F) obesity (E66); (G) renal failure (N17–N19); (H) sepsis (A40–A41). The line is the annual age-adjusted mortality rate and the shaded band is the 95% confidence interval, as reported by CDC WONDER. The renal failure series (panel G) is presented descriptively and its temporal trend is not interpreted [20]. ICD-10: International Classification of Diseases, Tenth Revision, CDC: Centers for Disease Control and Prevention, WONDER: Wide-ranging Online Data for Epidemiologic Research, CI: confidence interval.

Reference-year AAMRs for the co-occurring conditions moved in divergent directions over the study period (Table 3; Figure 5). The AAMR for gout-related deaths co-listing hypertension rose from 0.16 per 100,000 in 1999 to 0.35 in 2019 and 0.43 in 2024 (absolute change, +0.27), with a pronounced further increase during the pandemic years, peaking at 0.49 in 2021 (Figure 5). The AAMR co-listing diabetes mellitus similarly rose, from 0.12 in 1999 to 0.19 in 2019 and 0.20 in 2024 (absolute change, +0.08). In contrast, the AAMR co-listing ischemic heart disease was lower in 2024 (0.17) than in 1999 (0.26; absolute change, −0.09), while the AAMR co-listing heart failure was unchanged at the endpoints (0.15 in both 1999 and 2024). The AAMR co-listing renal failure was also lower in 2024 (0.08) than in 1999 (0.13). As with the other co-occurring conditions, this descriptive series was not modeled, and no inference is drawn from its trend; national renal-failure mortality has itself followed a complex, non-monotonic trajectory over this period [20]. Joinpoint regression was not applied to the co-occurring-condition series; these rates are presented descriptively.

Full annual data and model statistics are provided in Tables 4-7: annual observed and model-fitted AAMRs with standard errors by stratum (Table 4), joinpoint model fit statistics (Table 5), the Monte Carlo permutation test sequence used for model selection (Table 6), and annual AAMRs with 95% confidence intervals for the eight co-occurring conditions (Table 7).

**Table 4:** Annual observed age-adjusted mortality rate, joinpoint-fitted AAMR, and standard error for gout-related deaths (ICD-10 M10.0–M10.4, M10.9), United States, 1999–2024, by stratum Observed AAMRs are from CDC WONDER age-adjusted-rate output (reported to two decimals). Fitted AAMRs and standard errors are from NCI Joinpoint v6.0.1 (National Cancer Institute, Bethesda, Maryland) export files for each stratum. Obs.: observed, Fit.: fitted, SE: standard error, AAMR: age-adjusted mortality rate, ICD-10: International Classification of Diseases, Tenth Revision, CDC: Centers for Disease Control and Prevention, WONDER: Wide-ranging Online Data for Epidemiologic Research, NCI: National Cancer Institute, US: United States.

Year	Overall (US, all ages)			Male			Female			Non-Hispanic White			Non-Hispanic Black			Hispanic or Latino			Non-Hispanic Asian/Pacific Islander			Northeast			Midwest			South			West		
	Obs.	Fit.	SE	Obs.	Fit.	SE	Obs.	Fit.	SE	Obs.	Fit.	SE	Obs.	Fit.	SE	Obs.	Fit.	SE	Obs.	Fit.	SE	Obs.	Fit.	SE	Obs.	Fit.	SE	Obs.	Fit.	SE	Obs.	Fit.	SE
1999	0.57	0.57	0.0128	1.00	1.02	0.0332	0.32	0.32	0.0128	0.53	0.53	0.0153	1.22	1.23	0.0740	0.19	0.20	0.0434	1.20	1.19	0.1633	0.54	0.55	0.0306	0.63	0.64	0.0306	0.53	0.54	0.0230	0.60	0.62	0.0332
2000	0.60	0.58	0.0153	1.05	1.02	0.0332	0.33	0.32	0.0128	0.54	0.53	0.0153	1.26	1.23	0.0765	0.22	0.21	0.0459	1.23	1.17	0.1556	0.56	0.55	0.0306	0.68	0.66	0.0306	0.56	0.54	0.0230	0.65	0.62	0.0357
2001	0.58	0.58	0.0153	1.00	1.02	0.0306	0.31	0.32	0.0128	0.52	0.54	0.0153	1.18	1.23	0.0714	0.18	0.22	0.0383	1.16	1.15	0.1429	0.57	0.55	0.0306	0.66	0.68	0.0332	0.53	0.55	0.0230	0.60	0.62	0.0332
2002	0.58	0.59	0.0128	1.02	1.02	0.0332	0.32	0.33	0.0128	0.52	0.54	0.0128	1.25	1.22	0.0740	0.24	0.22	0.0459	1.18	1.13	0.1429	0.53	0.55	0.0306	0.73	0.70	0.0332	0.54	0.55	0.0230	0.55	0.62	0.0306
2003	0.61	0.60	0.0153	1.05	1.02	0.0306	0.34	0.33	0.0128	0.57	0.55	0.0153	1.23	1.22	0.0740	0.20	0.23	0.0408	1.08	1.11	0.1327	0.55	0.55	0.0306	0.71	0.73	0.0332	0.57	0.56	0.0230	0.62	0.63	0.0332
2004	0.59	0.60	0.0153	1.05	1.03	0.0306	0.31	0.34	0.0153	0.55	0.55	0.0153	1.14	1.22	0.0714	0.21	0.24	0.0408	0.93	1.09	0.1173	0.51	0.54	0.0306	0.70	0.75	0.0332	0.57	0.56	0.0230	0.64	0.63	0.0332
2005	0.61	0.61	0.0153	1.05	1.03	0.0306	0.34	0.34	0.0128	0.57	0.56	0.0153	1.28	1.22	0.0740	0.28	0.24	0.0434	1.05	1.07	0.1199	0.48	0.54	0.0281	0.81	0.78	0.0332	0.59	0.57	0.0230	0.63	0.63	0.0306
2006	0.63	0.62	0.0128	1.03	1.03	0.0306	0.37	0.34	0.0153	0.59	0.56	0.0153	1.18	1.22	0.0689	0.28	0.25	0.0459	1.16	1.05	0.1173	0.53	0.54	0.0281	0.79	0.81	0.0332	0.56	0.57	0.0230	0.68	0.63	0.0332
2007	0.61	0.62	0.0128	0.99	1.03	0.0281	0.36	0.35	0.0153	0.56	0.57	0.0128	1.33	1.22	0.0740	0.28	0.26	0.0408	0.96	1.03	0.1071	0.57	0.54	0.0281	0.80	0.78	0.0332	0.55	0.58	0.0230	0.56	0.63	0.0281
2008	0.61	0.63	0.0128	0.98	1.03	0.0281	0.34	0.35	0.0128	0.53	0.57	0.0128	1.20	1.22	0.0663	0.26	0.27	0.0408	0.96	1.01	0.1046	0.49	0.54	0.0281	0.72	0.76	0.0306	0.57	0.58	0.0230	0.62	0.63	0.0306
2009	0.62	0.63	0.0153	1.01	1.04	0.0281	0.36	0.36	0.0128	0.57	0.58	0.0153	1.16	1.22	0.0663	0.27	0.28	0.0383	0.94	0.99	0.0995	0.53	0.54	0.0281	0.76	0.74	0.0332	0.55	0.58	0.0230	0.65	0.63	0.0332
2010	0.65	0.64	0.0128	1.05	1.04	0.0281	0.36	0.36	0.0128	0.59	0.58	0.0153	1.27	1.22	0.0689	0.29	0.28	0.0408	1.08	0.97	0.1020	0.56	0.53	0.0281	0.77	0.72	0.0332	0.62	0.59	0.0230	0.64	0.64	0.0306
2011	0.66	0.65	0.0128	1.07	1.04	0.0281	0.35	0.36	0.0128	0.61	0.59	0.0153	1.21	1.22	0.0663	0.32	0.29	0.0408	0.91	0.92	0.0893	0.60	0.53	0.0306	0.71	0.70	0.0306	0.60	0.59	0.0230	0.72	0.64	0.0332
2012	0.64	0.63	0.0128	1.10	1.04	0.0281	0.36	0.35	0.0128	0.61	0.59	0.0153	1.15	1.16	0.0638	0.31	0.30	0.0383	0.83	0.87	0.0842	0.59	0.53	0.0306	0.74	0.69	0.0306	0.57	0.58	0.0204	0.72	0.64	0.0306
2013	0.60	0.61	0.0128	0.99	1.01	0.0255	0.32	0.34	0.0128	0.56	0.58	0.0153	1.15	1.10	0.0612	0.27	0.31	0.0357	0.82	0.82	0.0791	0.52	0.53	0.0281	0.64	0.67	0.0306	0.56	0.56	0.0204	0.68	0.64	0.0306
2014	0.60	0.59	0.0128	0.96	0.97	0.0255	0.33	0.33	0.0128	0.57	0.56	0.0128	1.00	1.05	0.0587	0.34	0.32	0.0383	0.75	0.77	0.0740	0.57	0.53	0.0281	0.67	0.65	0.0306	0.57	0.54	0.0230	0.58	0.64	0.0255
2015	0.57	0.58	0.0128	0.91	0.94	0.0255	0.34	0.32	0.0128	0.54	0.55	0.0153	0.96	0.99	0.0536	0.23	0.28	0.0306	0.73	0.73	0.0689	0.56	0.53	0.0281	0.61	0.63	0.0281	0.53	0.53	0.0204	0.65	0.64	0.0281
2016	0.56	0.56	0.0128	0.89	0.91	0.0230	0.31	0.31	0.0128	0.53	0.54	0.0128	0.92	0.94	0.0510	0.27	0.25	0.0332	0.72	0.69	0.0663	0.48	0.52	0.0255	0.58	0.62	0.0255	0.50	0.51	0.0204	0.68	0.64	0.0306
2017	0.54	0.55	0.0128	0.89	0.88	0.0230	0.29	0.30	0.0102	0.52	0.52	0.0128	0.93	0.90	0.0536	0.23	0.22	0.0281	0.72	0.65	0.0638	0.48	0.52	0.0255	0.58	0.60	0.0255	0.50	0.50	0.0179	0.62	0.65	0.0281
2018	0.55	0.53	0.0128	0.88	0.85	0.0230	0.29	0.30	0.0128	0.54	0.51	0.0128	0.86	0.85	0.0485	0.27	0.27	0.0306	0.58	0.62	0.0561	0.51	0.52	0.0255	0.55	0.58	0.0255	0.48	0.48	0.0179	0.63	0.65	0.0281
2019	0.55	0.59	0.0128	0.90	0.95	0.0230	0.28	0.31	0.0102	0.51	0.55	0.0128	0.93	0.98	0.0510	0.31	0.35	0.0306	0.62	0.68	0.0561	0.49	0.57	0.0255	0.52	0.57	0.0255	0.56	0.56	0.0204	0.59	0.70	0.0281
2020	0.69	0.65	0.0153	1.12	1.06	0.0255	0.34	0.32	0.0128	0.61	0.60	0.0153	1.23	1.13	0.0587	0.45	0.44	0.0357	0.77	0.75	0.0612	0.66	0.62	0.0281	0.63	0.55	0.0255	0.64	0.64	0.0204	0.76	0.76	0.0281
2021	0.73	0.72	0.0128	1.18	1.18	0.0255	0.37	0.32	0.0128	0.67	0.65	0.0153	1.33	1.30	0.0612	0.43	0.40	0.0357	0.83	0.83	0.0663	0.68	0.67	0.0306	0.57	0.54	0.0281	0.77	0.73	0.0230	0.85	0.83	0.0306
2022	0.64	0.68	0.0128	1.08	1.11	0.0255	0.32	0.33	0.0102	0.62	0.63	0.0153	1.08	1.18	0.0536	0.35	0.37	0.0306	0.78	0.74	0.0612	0.61	0.63	0.0281	0.51	0.52	0.0255	0.65	0.71	0.0204	0.81	0.79	0.0306
2023	0.64	0.65	0.0128	1.03	1.05	0.0230	0.32	0.33	0.0128	0.61	0.62	0.0128	1.04	1.08	0.0536	0.36	0.34	0.0332	0.59	0.67	0.0536	0.59	0.60	0.0281	0.49	0.51	0.0255	0.68	0.68	0.0204	0.73	0.75	0.0281
2024	0.63	0.62	0.0102	1.02	0.99	0.0255	0.33	0.34	0.0128	0.61	0.60	0.0153	1.04	0.98	0.0536	0.30	0.31	0.0281	0.63	0.60	0.0536	0.58	0.57	0.0281	0.50	0.50	0.0230	0.68	0.66	0.0204	0.71	0.71	0.0281

**Table 5:** Joinpoint model fit statistics for gout-related mortality (ICD-10 M10.0–M10.4, M10.9) by stratum, United States, 1999–2024. NCI Joinpoint Regression Program v6.0.1; log-linear model; uncorrelated errors; grid search; parametric 95% confidence intervals; seed 7160 * indicates APC significantly different from zero at α = 0.05. Joinpoint year 95% CIs, parameter counts, residual degrees of freedom, SSE, and MSE are from the NCI Joinpoint Model Estimates export for each stratum. See Table 6 for the Monte Carlo permutation test sequence. US: United States, CI: confidence interval, obs.: observations, DF: degrees of freedom, SSE: sum of squared errors, MSE: mean squared error, APC: annual percent change, NCI: National Cancer Institute, ICD-10: International Classification of Diseases, Tenth Revision.

Stratum	Final model (no. of joinpoints)	Joinpoint year(s) (95% CI)	Obs., N	No. of parameters	Residual DF	SSE	MSE	Segment	Intercept	Slope (log-rate/year)	APC%
Overall (US, all ages)	3	2011 (2008-2014); 2018 (2016-2019); 2021 (2020-2022)	26	8	18	44.0892	2.4494	Seg 0: 1999-2011	-21.0548	0.010254	+1.03*
								Seg 1: 2011-2018	56.9899	-0.028555	−2.82*
								Seg 2: 2018-2021	-201.6704	0.099621	+10.48*
								Seg 3: 2021-2024	98.1249	-0.048719	−4.76*
Male	3	2012 (2009-2015); 2018 (2016-2019); 2021 (2020-2022)	26	8	18	34.9123	1.9396	Seg 0: 1999-2012	-3.6721	0.001845	+0.18
								Seg 1: 2012-2018	68.5223	-0.034037	−3.35*
								Seg 2: 2018-2021	-220.9994	0.109433	+11.56*
								Seg 3: 2021-2024	116.7585	-0.057691	−5.61*
Female	2	2011 (2004-2016); 2017 (2012-2020)	26	6	20	48.9993	2.4500	Seg 0: 1999-2011	-23.3979	0.011131	+1.12*
								Seg 1: 2011-2017	65.3882	-0.033019	−3.25
								Seg 2: 2017-2024	-40.4712	0.019464	+1.97
Non-Hispanic White	3	2012 (2008-2016); 2018 (2015-2019); 2021 (2019-2022)	26	8	18	47.2490	2.6249	Seg 0: 1999-2012	-19.6342	0.009500	+0.95*
								Seg 1: 2012-2018	51.6207	-0.025915	−2.56*
								Seg 2: 2018-2021	-166.4388	0.082142	+8.56
								Seg 3: 2021-2024	54.9085	-0.027381	−2.70
Non-Hispanic Black	3	2011 (2006-2014); 2018 (2016-2019); 2021 (2020-2022)	26	8	18	18.6255	1.0347	Seg 0: 1999-2011	1.0332	-0.000415	−0.04
								Seg 1: 2011-2018	103.6933	-0.051464	−5.02*
								Seg 2: 2018-2021	-285.5404	0.141417	+15.19*
								Seg 3: 2021-2024	189.7613	-0.093765	−8.95*
Hispanic or Latino	3	2014 (2007-2015); 2017 (2016-2019); 2020 (2019-2022)	26	8	18	11.4363	0.6354	Seg 0: 1999-2014	-61.8586	0.030150	+3.06*
								Seg 1: 2014-2017	266.3115	-0.132794	−12.44
								Seg 2: 2017-2020	-482.5643	0.238488	+26.93*
								Seg 3: 2020-2024	176.2927	-0.087679	−8.39*
Non-Hispanic Asian/Pacific Islander	3	2010 (2001-2016); 2018 (2016-2019); 2021 (2020-2022)	26	8	18	10.8234	0.6013	Seg 0: 1999-2010	37.5543	-0.018698	−1.85*
								Seg 1: 2010-2018	114.6084	-0.057034	−5.54*
								Seg 2: 2018-2021	-199.7293	0.098733	+10.38
								Seg 3: 2021-2024	218.4774	-0.108197	−10.26*
Northeast	2	2018 (2015-2019); 2021 (2019-2022)	26	6	20	42.3539	2.1177	Seg 0: 1999-2018	5.7859	-0.003191	−0.32
								Seg 1: 2018-2021	-171.4545	0.084639	+8.83
								Seg 2: 2021-2024	113.9528	-0.056582	−5.50
Midwest	1	2006 (2004-2009)	26	4	22	35.0630	1.5938	Seg 0: 1999-2006	-68.2214	0.033901	+3.45*
								Seg 1: 2006-2024	53.7952	-0.026924	−2.66*
South	3	2011 (2007-2016); 2018 (2017-2019); 2021 (2020-2022)	26	8	18	21.8073	1.2115	Seg 0: 1999-2011	-17.5874	0.008487	+0.85*
								Seg 1: 2011-2018	58.7170	-0.029456	−2.90*
								Seg 2: 2018-2021	-279.4474	0.138118	+14.81*
								Seg 3: 2021-2024	69.7454	-0.034664	−3.41
West	2	2018 (2015-2019); 2021 (2020-2022)	26	6	20	55.1355	2.7568	Seg 0: 1999-2018	-4.9277	0.002226	+0.22
								Seg 1: 2018-2021	-168.0223	0.083046	+8.66
								Seg 2: 2021-2024	107.4342	-0.053251	−5.19

**Table 6:** Monte Carlo permutation test sequence used for joinpoint model selection, by stratum. NCI Joinpoint v6.0.1; 4,499 permutations per test; sequential testing with Bonferroni-adjusted significance levels The "Selected" column shows the number of joinpoints chosen by the testing procedure at each step; the final selected model corresponds to the joinpoint count selected at the last test. US: United States, H₀: null hypothesis, H₁: alternative hypothesis, JPs: joinpoints, DF: degrees of freedom, NCI: National Cancer Institute.

Stratum	Test #	H₀ (# JPs)	H₁ (# JPs)	Selected	Num. DF	Den. DF	# permutations	P-value	Significance level
Overall (US, all ages)	0	0	4	4	8	16	4500	<0.001	0.0125
	1	1	4	4	6	16	4500	<0.001	0.0167
	2	2	4	4	4	16	4500	0.001	0.0250
	3	3	4	3	2	16	4500	0.833	0.0500
Male	0	0	4	4	8	16	4500	<0.001	0.0125
	1	1	4	4	6	16	4500	<0.001	0.0167
	2	2	4	4	4	16	4500	0.002	0.0250
	3	3	4	3	2	16	4500	0.425	0.0500
Female	0	0	4	4	8	16	4500	0.002	0.0125
	1	1	4	4	6	16	4500	0.004	0.0167
	2	2	4	2	4	16	4500	0.055	0.0250
	3	2	3	2	2	18	4500	0.036	0.0250
Non-Hispanic White	0	0	4	4	8	16	4500	<0.001	0.0125
	1	1	4	4	6	16	4500	0.001	0.0167
	2	2	4	2	4	16	4500	0.032	0.0250
	3	2	3	3	2	18	4500	0.017	0.0250
Non-Hispanic Black	0	0	4	4	8	16	4500	<0.001	0.0125
	1	1	4	4	6	16	4500	<0.001	0.0167
	2	2	4	4	4	16	4500	<0.001	0.0250
	3	3	4	3	2	16	4500	0.902	0.0500
Hispanic or Latino	0	0	4	4	8	16	4500	<0.001	0.0125
	1	1	4	4	6	16	4500	<0.001	0.0167
	2	2	4	4	4	16	4500	0.002	0.0250
	3	3	4	3	2	16	4500	0.498	0.0500
Non-Hispanic Asian/Pacific Islander	0	0	4	4	8	16	4500	0.006	0.0125
	1	1	4	4	6	16	4500	0.004	0.0167
	2	2	4	2	4	16	4500	0.086	0.0250
	3	2	3	3	2	18	4500	0.020	0.0250
Northeast	0	0	4	4	8	16	4500	0.004	0.0125
	1	1	4	4	6	16	4500	0.005	0.0167
	2	2	4	2	4	16	4500	0.056	0.0250
	3	2	3	2	2	18	4500	0.071	0.0250
Midwest	0	0	4	4	8	16	4500	<0.001	0.0125
	1	1	4	1	6	16	4500	0.276	0.0167
	2	1	3	1	4	18	4500	0.552	0.0167
	3	1	2	1	2	20	4500	0.922	0.0167
South	0	0	4	4	8	16	4500	<0.001	0.0125
	1	1	4	4	6	16	4500	<0.001	0.0167
	2	2	4	4	4	16	4500	<0.001	0.0250
	3	3	4	3	2	16	4500	0.889	0.0500
West	0	0	4	0	8	16	4500	0.018	0.0125
	1	0	3	3	6	18	4500	0.012	0.0125
	2	1	3	3	4	18	4500	0.006	0.0167
	3	2	3	2	2	18	4500	0.088	0.0250

**Table 7:** Annual age-adjusted mortality rates (per 100,000) for co-occurring causes of death listed with gout (ICD-10 M10.0–M10.4, M10.9) on U.S. death certificates, 1999–2024 AAMRs and 95% CIs (LCL, UCL) are as reported by CDC WONDER, age-standardized to the 2000 US standard population. Data combine the 1999–2020 Bridged-Race and 2021–2024 Single-Race files. AAMR: age-adjusted mortality rate, LCL: lower confidence limit, UCL: upper confidence limit, CI: confidence interval, ICD-10: International Classification of Diseases, Tenth Revision, US: United States, CDC: Centers for Disease Control and Prevention, WONDER: Wide-ranging Online Data for Epidemiologic Research.

Year	Hypertension (I10-I15)			Ischemic heart disease (I20-I25)			Heart failure (I50)			Cerebrovascular disease (I60-I69)			Diabetes mellitus (E10-E14)			Obesity (E66)			Renal failure (N17-N19)			Sepsis (A40-A41)		
	AAMR	LCL	UCL	AAMR	LCL	UCL	AAMR	LCL	UCL	AAMR	LCL	UCL	AAMR	LCL	UCL	AAMR	LCL	UCL	AAMR	LCL	UCL	AAMR	LCL	UCL
1999	0.16	0.14	0.17	0.26	0.24	0.28	0.15	0.14	0.17	0.08	0.07	0.09	0.12	0.10	0.13	0.02	0.01	0.03	0.13	0.12	0.15	0.02	0.02	0.03
2000	0.30	0.28	0.32	0.27	0.25	0.29	0.17	0.16	0.19	0.09	0.08	0.10	0.12	0.11	0.14	0.01	0.01	0.02	0.16	0.15	0.18	0.03	0.02	0.04
2001	0.29	0.27	0.31	0.28	0.26	0.30	0.16	0.14	0.17	0.09	0.08	0.10	0.13	0.12	0.15	0.01	0.01	0.02	0.14	0.13	0.16	0.04	0.03	0.05
2002	0.31	0.28	0.33	0.25	0.23	0.27	0.16	0.15	0.18	0.09	0.08	0.10	0.14	0.12	0.15	0.01	0.01	0.02	0.16	0.14	0.17	0.03	0.02	0.04
2003	0.33	0.31	0.35	0.26	0.24	0.28	0.16	0.15	0.18	0.08	0.07	0.09	0.15	0.13	0.16	0.02	0.02	0.03	0.17	0.15	0.18	0.02	0.02	0.03
2004	0.32	0.30	0.34	0.24	0.23	0.26	0.18	0.16	0.19	0.08	0.07	0.09	0.15	0.14	0.17	0.02	0.02	0.03	0.16	0.15	0.17	0.04	0.03	0.04
2005	0.36	0.33	0.38	0.24	0.22	0.25	0.18	0.16	0.19	0.09	0.08	0.10	0.15	0.13	0.16	0.01	0.01	0.02	0.18	0.16	0.19	0.06	0.05	0.06
2006	0.34	0.32	0.36	0.25	0.23	0.26	0.18	0.16	0.19	0.09	0.08	0.10	0.17	0.16	0.19	0.01	0.01	0.02	0.18	0.17	0.20	0.04	0.03	0.04
2007	0.37	0.35	0.39	0.25	0.23	0.26	0.17	0.15	0.18	0.07	0.06	0.08	0.17	0.15	0.18	0.02	0.02	0.03	0.15	0.14	0.17	0.03	0.02	0.04
2008	0.35	0.33	0.37	0.25	0.23	0.26	0.16	0.14	0.17	0.08	0.07	0.09	0.18	0.16	0.19	0.02	0.01	0.02	0.15	0.13	0.16	0.03	0.02	0.04
2009	0.37	0.35	0.39	0.25	0.24	0.27	0.16	0.15	0.18	0.07	0.06	0.08	0.19	0.17	0.20	0.02	0.02	0.03	0.14	0.13	0.16	0.04	0.03	0.05
2010	0.38	0.36	0.41	0.25	0.23	0.27	0.17	0.15	0.18	0.08	0.07	0.09	0.20	0.19	0.22	0.03	0.02	0.04	0.14	0.13	0.15	0.04	0.03	0.04
2011	0.40	0.38	0.42	0.26	0.24	0.27	0.17	0.15	0.18	0.07	0.06	0.08	0.17	0.16	0.19	0.03	0.02	0.03	0.20	0.18	0.21	0.04	0.03	0.05
2012	0.41	0.39	0.43	0.25	0.24	0.27	0.18	0.17	0.19	0.07	0.06	0.08	0.18	0.17	0.20	0.02	0.01	0.02	0.20	0.19	0.22	0.04	0.03	0.05
2013	0.38	0.36	0.40	0.22	0.21	0.24	0.15	0.14	0.16	0.07	0.06	0.07	0.19	0.17	0.20	0.02	0.02	0.03	0.08	0.07	0.09	0.04	0.03	0.04
2014	0.37	0.35	0.39	0.22	0.20	0.23	0.16	0.14	0.17	0.06	0.05	0.07	0.19	0.17	0.20	0.03	0.02	0.03	0.08	0.07	0.09	0.04	0.03	0.05
2015	0.38	0.36	0.40	0.21	0.19	0.22	0.15	0.14	0.17	0.07	0.06	0.08	0.18	0.17	0.20	0.02	0.02	0.03	0.06	0.05	0.07	0.04	0.03	0.05
2016	0.36	0.34	0.38	0.17	0.16	0.19	0.15	0.14	0.16	0.06	0.05	0.06	0.18	0.16	0.19	0.02	0.02	0.03	0.05	0.04	0.06	0.03	0.02	0.04
2017	0.36	0.34	0.38	0.19	0.18	0.21	0.15	0.13	0.16	0.06	0.05	0.06	0.17	0.16	0.18	0.02	0.02	0.03	0.06	0.05	0.06	0.03	0.02	0.03
2018	0.38	0.36	0.40	0.17	0.16	0.19	0.14	0.13	0.15	0.06	0.05	0.06	0.16	0.15	0.18	0.03	0.02	0.03	0.06	0.05	0.06	0.02	0.02	0.03
2019	0.35	0.33	0.37	0.19	0.17	0.20	0.15	0.14	0.17	0.05	0.05	0.06	0.19	0.18	0.21	0.03	0.02	0.03	0.05	0.04	0.06	0.04	0.03	0.05
2020	0.46	0.44	0.48	0.20	0.18	0.21	0.16	0.14	0.17	0.07	0.07	0.08	0.22	0.20	0.23	0.05	0.04	0.06	0.08	0.07	0.09	0.04	0.04	0.05
2021	0.49	0.47	0.52	0.21	0.20	0.23	0.17	0.16	0.18	0.09	0.08	0.10	0.23	0.22	0.25	0.06	0.05	0.07	0.09	0.08	0.10	0.06	0.05	0.06
2022	0.46	0.43	0.48	0.21	0.20	0.23	0.15	0.14	0.16	0.07	0.06	0.08	0.20	0.19	0.21	0.05	0.04	0.06	0.08	0.07	0.08	0.04	0.03	0.05
2023	0.44	0.42	0.46	0.18	0.17	0.20	0.15	0.14	0.17	0.08	0.07	0.09	0.19	0.17	0.20	0.04	0.03	0.05	0.07	0.07	0.08	0.05	0.05	0.06
2024	0.43	0.41	0.45	0.17	0.16	0.19	0.15	0.13	0.16	0.07	0.06	0.08	0.20	0.19	0.22	0.05	0.04	0.06	0.08	0.07	0.08	0.05	0.04	0.05

## Discussion

We characterized gout-related mortality in the United States from 1999 through 2024 using national multiple-cause death certificate data. To our knowledge, no prior study has applied joinpoint regression to US national death certificate data to describe population-level gout-related mortality across demographic and regional strata over a period spanning the pre-pandemic decade and the COVID-19 era. The overall age-adjusted rate changed little across the 26 years, but that stability was superficial. The trend moved through four distinct phases, accelerated sharply in the years surrounding the pandemic, and varied considerably by sex, race and Hispanic origin, region, and urbanization. Deaths were concentrated at older ages and carried a consistent cardiometabolic-renal comorbidity signature. Read together, these patterns are consistent with gout-related mortality reflecting the comorbidity burden that accompanies the disease: on death certificates, gout is co-listed with cardiometabolic and renal conditions far more often than it is recorded as the proximate cause. Because the design is descriptive, this association does not establish that comorbidities rather than gout drive the observed trends.

This observed picture differs from the modeled global burden. Estimates derived from the Global Burden of Disease framework show rising gout prevalence and burden over recent decades [3], and US prevalence has remained substantial and stable [2], yet the observed national death rate we measured was essentially flat. The discrepancy is expected. Gout is seldom the condition that ends a life; it appears on the certificate alongside the cardiovascular, metabolic, and renal diseases that do, and modeled mortality estimates infer gout deaths indirectly rather than counting them. The early rise from 1999 to 2011 is consistent with the accumulating burden of these comorbidities (over the same interval, the rates of gout deaths co-listing hypertension and diabetes climbed steadily), and may partly reflect changes in death certificate reporting practices, which have been shown to inflate multiple-cause mortality trends for contributing conditions [21]. The decline from 2011 to 2018 coincides with a steady fall over the same interval in the rate of gout deaths co-listing ischemic heart disease (Table 7), itself consistent with the long-term national decline in ischemic heart disease mortality [22]. Across both phases, the gout-related rate tracked the net comorbidity burden and its shifting composition rather than the prevalence of gout.

The most pronounced feature was the acceleration the model placed between 2018 and 2021. The inflection point precedes the pandemic, but the steep rise within that segment was concentrated in the pandemic years: the observed rate held at 0.55 per 100,000 in 2018 and 2019, then rose to 0.69 in 2020 and 0.73 in 2021. Several lines of external evidence are consistent with a pandemic-related contribution, though our data cannot test it directly. People with gout are an older, heavily multimorbid population, and our decedents were no exception, with more than two-thirds aged 75 years or older. US excess mortality in 2020 was concentrated in exactly this age range and fell disproportionately on Black and Hispanic populations [23]. People with gout specifically carried an elevated risk of COVID-19 death in population-based data [24], a risk attributed to the cardiometabolic and renal comorbidities that cluster with the disease [25]. Gout care was disrupted at the same time, with patients reporting difficulty obtaining medications and reaching clinicians [26]. Taken together, these factors offer a plausible explanation for the timing of the rise, namely that gout-related mortality increased as part of the broader pandemic-era rise in deaths among multimorbid older adults, with gout recorded as a contributing condition rather than the proximate cause. We did not test COVID-19 co-listing on gout-related certificates, and this design cannot separate deaths caused directly by SARS-CoV-2 from those reflecting disrupted care, delayed treatment, or shifts in death certificate practice; the pandemic-era association is therefore hypothesized from timing and external literature rather than demonstrated here. The decline after 2021 is consistent with the waning of pandemic mortality as vaccination, accumulated immunity, and restored access to care took hold. Joinpoint regression dates these inflections; it cannot attribute them, a limitation we return to below.

The demographic patterns align with what is known about who develops gout and who is treated adequately for it. Mortality was roughly twice as high in men as in women, mirroring the male predominance of gout, which narrows in later life as the sex difference in prevalence diminishes after menopause [27]. The absolute rates are not the whole story: population-based data indicate that the relative excess in COVID-19 death associated with gout was greater in women than in men [24], a reminder that absolute and relative measures can diverge.

Differences by race and Hispanic origin were larger and more informative. Non-Hispanic Black decedents had the highest mortality, consistent with a higher prevalence of gout, a heavier burden of the hypertensive and renal comorbidities that accompany it, and documented disparities in gout care, including lower receipt of urate-lowering therapy and markedly higher rates of gout-related emergency and inpatient care [27,28]. Hispanic decedents had the lowest overall rate, which matches their lower reported prevalence of gout [28], yet showed the steepest single-segment acceleration of any group during the pandemic years. That juxtaposition is coherent rather than contradictory: a low baseline reflects lower disease frequency, while the sharp pandemic-era rise reflects the disproportionate mortality that Hispanic and Black communities experienced in 2020 [23]. The non-Hispanic Asian or Pacific Islander group ran counter to every other pattern, declining significantly across the full period. This is the least certain of our findings. It conflicts with the higher gout risk reported for Asian and Native Hawaiian populations [28], and it rests on a category whose definition changed between the two data vintages and whose recent rates we approximated from single-race Asian decedents alone. We therefore present this decline as a finding to be confirmed rather than interpreted and note that its magnitude will change once the combined rates are recomputed.

Geographic and urban-rural patterns were harder to interpret and are best treated as hypothesis-generating. The South showed the same pandemic-era surge seen nationally, whereas the Midwest followed a different course entirely, with a single inflection in 2006 and a long, significant decline thereafter that owed nothing to the pandemic. Neither the Northeast nor the West showed a statistically significant segment trend. A clearer signal came from urbanization: the crude mortality rate rose steadily from large central metropolitan areas to the most rural counties, where it was roughly twice as high. This gradient may reflect the higher prevalence of cardiometabolic risk factors and more limited access to specialty care in rural areas, though our ecological data cannot identify the responsible mechanisms.

The comorbidity profile is the thread that connects these patterns. Hypertension accompanied gout on more than three in five death certificates, followed by ischemic heart disease, diabetes, heart failure, and renal failure, the cardiometabolic-renal cluster that characterizes patients with gout, in whom cohort studies have consistently documented excess all-cause and cardiovascular mortality [4,5,7]. The co-occurring conditions did not move together. Rates of gout deaths co-listing hypertension and diabetes rose over the study period, while those co-listing ischemic heart disease fell, the latter mirroring the long-term national decline in ischemic heart disease mortality [22]. The frequent co-listing of renal failure underscores the close, bidirectional relationship between gout and kidney disease. We draw no inference from the temporal trend in this descriptive series, particularly given the complex, non-monotonic national trajectory of renal failure mortality itself over this period [20]. These findings extend the cohort literature, which has shown that the excess mortality associated with gout reaches beyond cardiovascular disease to genitourinary and digestive causes [6], to the level of the whole US decedent population.

The clinical implication follows from the comorbidity picture. Effective, guideline-concordant treatment for gout exists; the American College of Rheumatology recommends a treat-to-target urate-lowering strategy that controls the disease in most patients [29]. Yet gout-related mortality persisted across the study period, surged in a high-risk multimorbid population during the pandemic, and fell hardest on groups already underserved by gout care. The opportunity therefore lies less in new therapy than in delivering existing care consistently, integrated with management of the cardiometabolic and renal conditions that drive these deaths, and directed toward the populations and settings where the burden is greatest. The pandemic experience also marks multimorbid patients with gout as a group warranting specific attention when health systems are strained.

This study has several strengths. It provides an observed, death-certificate-based account of gout-related mortality rather than a modeled estimate, spans 26 years including the pandemic, and uses the multiple-cause file to capture gout where it appears on the certificate. Joinpoint regression localized inflection points that the flat full-period summary concealed.

The limitations bear equal weight. Death-certificate data are subject to misclassification and to variation in certifier practice, and the multiple-cause approach establishes that gout was recorded alongside another cause, not that it contributed to death. The analysis is ecological and cannot support individual-level or causal inference, and it cannot separate deaths caused directly by SARS-CoV-2 from those reflecting disrupted care; a future analysis of COVID-19 co-listing on gout-related certificates would help test the first mechanism. The permutation testing controls the joinpoint-selection error rate within each stratum's sequential model but does not correct for the 11 stratified models fitted across the study; stratum-specific findings, particularly those based on small numbers of deaths, should therefore be regarded as hypothesis-generating rather than confirmatory. This caution applies most directly to the Hispanic 2017-2020 segment, whose large estimated increase (APC +26.93%) rests on a small denominator and a correspondingly wide confidence interval (95% CI, +3.01 to +56.41) and should not be overinterpreted. The transition from bridged-race to single-race classification at the 2020/2021 boundary, with differing population denominators on either side, complicates interpretation of the pandemic-era segments, although the surge begins in 2020 within the earlier vintage. The non-Hispanic Asian or Pacific Islander trend rates were approximated from single-race Asian decedents and exclude Pacific Islanders, who are retained in the count; this modestly understates the most recent rates and should be reconciled. Cells with fewer than 10 deaths were suppressed, constraining the youngest age strata and the sparsest demographic combinations. Death certificates also carry no information on gout disease severity, serum urate concentration, urate-lowering therapy or adherence, individual-level comorbidity burden, or socioeconomic status, so the contribution of these factors to the observed trends cannot be assessed. Finally, the renal comorbidity trend is presented descriptively and is not interpreted, given the complex national trajectory of renal failure mortality.

## Conclusions

Gout-related mortality in the United States was stable on average between 1999 and 2024 but moved through four distinct phases, accelerated in the years surrounding the COVID-19 pandemic, and varied sharply by sex, race and Hispanic origin, region, and urbanization. The trajectory is best understood not as a measure of gout's own lethality but as a barometer of the cardiometabolic and renal multimorbidity that accompanies it. Reducing this mortality will depend less on new treatment than on delivering established gout and comorbidity care consistently and on reaching the older, multimorbid, and underserved populations in whom these deaths are concentrated.
